# Laboratory Calibration of a Field Imaging Spectrometer System

**DOI:** 10.3390/s110302408

**Published:** 2011-02-25

**Authors:** Lifu Zhang, Changping Huang, Taixia Wu, Feizhou Zhang, Qingxi Tong

**Affiliations:** 1 School of Earth and Space Sciences, Peking University, 100871 Beijing, China; E-Mail: zhanglf@pku.edu.cn; 2 The State Key Laboratory of Remote Sensing Sciences, Institute of Remote Sensing Applications, Chinese Academy of Sciences, 100101 Beijing, China; E-Mails: hcp2qq@163.com (C.-P.H.); wutaixia@126.com (T.-X.W.); tqxi@263.net (Q.-X.T.); 3 Graduate University of Chinese Academy of Sciences, 100039 Beijing, China

**Keywords:** hyperspectral remote sensing, imaging spectrometer, field imaging spectrometer system, FISS, calibration

## Abstract

A new Field Imaging Spectrometer System (FISS) based on a cooling area CCD was developed. This paper describes the imaging principle, structural design, and main parameters of the FISS sensor. The FISS was spectrally calibrated with a double grating monochromator to determine the center wavelength and FWHM of each band. Calibration results showed that the spectral range of the FISS system is 437–902 nm, the number of channels is 344 and the spectral resolution of each channel is better than 5 nm. An integrating sphere was used to achieve absolute radiometric calibration of the FISS with less than 5% calibration error for each band. There are 215 channels with signal to noise ratios (SNRs) greater than 500 (62.5% of the bands). The results demonstrated that the FISS has achieved high performance that assures the feasibility of its practical use in various fields.

## 1. Introduction

Imaging spectrometry combines traditional 2-D imaging remote sensing technology and spectroscopy [1–3], allowing for the acquisition of both images and spectra of objects. The emergence of imaging spectrometers has resolved the historical problems of “non-spectral imaging” and “non-imaging spectra” in traditional scientific fields [2,4]. Since its development, imaging spectrometry has been used in a wide range of fields for specific target detection [5,6], precise classification [2,7,8], and the quantitative retrieval of biochemical or biophysical parameters [9–11]. Unfortunately, data acquired by airborne or spaceborne imaging spectrometers can only be used to monitor objects on a macroscopic scale, with a sparse ground resolution of a few meters to several kilometers [12]. As they are affected by uncontrollable factors, including observation scale, angle, and complex backgrounds, the spectra extracted from both airborne and spaceborne images are rarely pure. They are often called mixed spectra [13], and bring a certain degree of bias to the analysis. To address these problems, field imaging spectrometry has been developed. Since 1990, many countries, including the USA, Japan, and Europe, have launched a series of mature field imaging spectrometers which have been successfully applied in agriculture [14], food monitoring [15,16], vegetation observations [17], geological mapping [18], and other fields [19,20]. The unique advantages of field imaging spectrometers have catalyzed the development of field imaging spectroscopy and promoted further improvements in both field spectral measurements and aviation imaging spectrometry.

Although China’s aviation imaging spectrometry is relatively mature [4,21], the development of ground-based imaging spectrometry has only just begun, and few applications using such equipment have been reported in China. Recently, to narrow the gap between China and the countries mentioned above, we have developed a new field imaging spectrometer system (FISS), based on the aviation push-broom imaging spectrometer (PHI) [22], self-developed in China at the Institute of Remote Sensing Applications and the Shanghai Institute of Technical Physics, as part of the Chinese Academy of Sciences. For indoor or outdoor measurements, the FISS instrument can obtain high-resolution images of targets (spatial resolution up to the cm or mm scale) and extract a complete spectrum of every pixel from images obtained in the wavelength region covered. Our experiments [23–25] using FISS confirmed that it can greatly improve the efficiency of field spectral measurements, provide information for the analysis of structural spectra, decompose mixture spectra, and extract pure spectra. Compared to those produced by traditional field spectrometers (e.g., ASD FieldSpec), the spectra derived by FISS may be considered pure. They are helpful for studying the mixing mechanism of surface units and analyzing spectral mixtures over varying spatial scales [24].

The data acquired by the FISS instrument are A/D converter counts (Digital Number, DN), in arbitrary units mainly defined by the integration time and solar lamp intensity [26]. If DNs are not further converted to reflectance or absorbance, they have no physical meaning. Therefore, to make quantitative studies of surface features, accurate radiometric and spectral calibration of the data must be performed [26–29]. The methodologies and measurements for sensor calibration have been studied in detail, and can often be grouped into three stages. These are laboratory calibration prior to launch, in-orbit/in-flight calibration, and vicarious or ground-look calibration [30–34]. As our FISS instrument is mainly used for field measurements, this paper describes only the first stage. There are two major tasks in laboratory calibration. The first is spectral calibration, which consists of determining the spectral response function for each band through the centroid wavelength and spectral resolution. It is calculated as the full-width at half-maximum (FWHM) of the spectral response function for each band. The second task is radiometric calibration, which consists of resolving the conversion coefficients between the digital number output from a sensor and the uniform-radiance field at its entrance pupil, which is routinely assumed to be a linear sensor system.

This paper introduces China’s first field imaging spectrometer, FISS. Its imaging principles, structural design, and main parameters are described. Spectral and radiometric calibration were performed in the precision optical laboratory of the Anhui Institute of Optics and Fine Mechanics, Chinese Academy of Sciences. The sensor’s signal to noise ratio (SNR) was also precisely measured. This work will be of crucial importance in boosting the development of field imaging spectroscopy in China.

## 2. FISS System Overview

### 2.1. Basic Design Principles

The FISS development process drew inspiration from that of the PHI. We focused on improvement or redesign of the imaging system, optical splitting system, and control software. The imaging system is similar to that of PHI [4,35]; the direction along the slit forms a spatial line image, while that perpendicular to the slit measures the spectrum for each line pixel made by the dispersion component. A second spatial dimension is covered by the scanning mirror.

**Figure 1.**
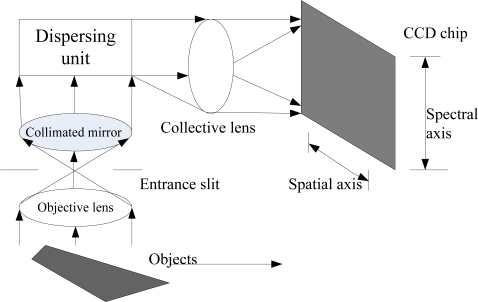
Basic principle of the FISS instrument.

**Figure 2.**
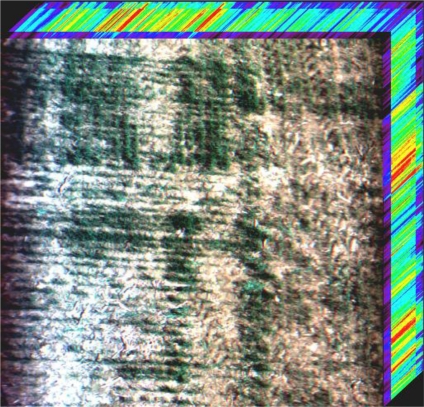
Vegetation image cube acquired by the FISS. The hyperspectral image was taken by FISS fixed in an elevated car, which was located 30 m above a wheat field in the Xiaotangshan National Demonstration Base for Precision Agriculture Research, Beijing, China.

Figure 1 shows the imaging principle of the FISS. The front optics image the object line onto the entrance slit plate, and then successively pass light through a collimating mirror, a dispersing unit in which the incident radiation is spread according to its wavelength in the vertical direction. Finally, a collective lens forms an image on the CCD chip. In the image, the spectra of the object line are represented by values found in the rows (parallel to the slit, called the spatial axis), while radiation within a narrow spectral band received from the line is found in the columns (dispersion direction, called the spectral axis). For each object line, the CCD can generate a spectral-spatial image, and together with the pendulum sweeping of the scan mirror within a certain angle and record rate, spectral data can be recorded continuously. The result is an image cube, as shown in Figure 2.

### 2.2. Structural Design

**Figure 3.**
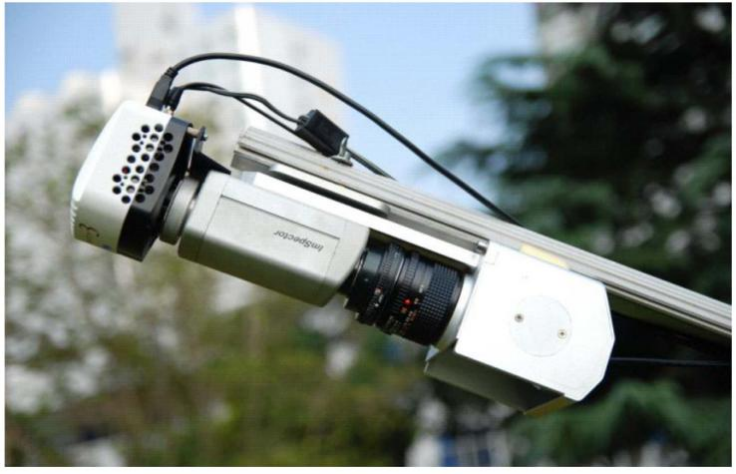
Photograph of the FISS optomechanical subsystem: from upper left to lower right of the enclosed optomechanical subsystem are the CCD camera, which is built with area array detectors and a cooling device, the dispersing unit with a “prism-grating-prism” (PGP) element, the objective lens, and the scan mirror. The latter is attached to a stepper motor.

The FISS consists of three main parts: the computer subsystem, the optomechanical subsystem, and the electronic subsystem. To achieve high performance, all three subsystems are important. However, the essential part of FISS is the optomechanical subsystem, which incorporates the scan mirror, an objective lens, a dispersing unit, and a CCD camera. Therefore, it performs scanning, imaging, dispersion, photoelectric conversion, A/D conversion, and other important functions. Figure 3 shows the actual FISS optomechanical subsystem.

As shown in Figure 3, the scan mirror unit, which is composed of an elliptical reflecting mirror, a stepper motor, and a mechanical framework, is driven by the stepper motor. The scan motor swings back and forth within a certain angle to cover one spatial dimension of an object. Through the objective lens, the surface features are clearly imaged onto the entrance slit plate on the focal plane of the dispersion unit. Using “prism-grating-prism” (PGP) spectrographs [36], the dispersing unit disperses the light beam from the entrance slit, and the rays of different wavelengths are then separated by the PGP-element and captured by the focal plane of the CCD camera. In the CCD chip (Model INFINITY3-1), the radiation is converted into proportional electrical signals, which largely determine the clarity of the images obtained.

The power supply and motor control circuits are incorporated into the electronic subsystem to ensure successful FISS operation. Finally, the raw imaging data and other ancillary data can be transmitted to a computer subsystem (a portable laptop computer, including control software and some basic data processing/analysis programs) by data transmission lines with high-speed USB2.0 interfaces, to enable real-time monitoring and data storage.

**Figure 4.**
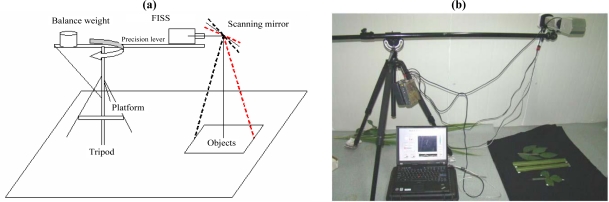
**(a)** Schematic and **(b)** photograph of FISS field measurements based on the multi-use platform.

In addition to the parts described above, a multi-use platform was specially designed to facilitate field measurements. It was intended to be sturdy and durable, easy to dismantle, compact, mobile, flexible, and easy to set up. The multi-use platform consists of a tripod and a precision lever, used to carry the optomechanical and electronic subsystems. The length and angle of the lever are controllable so that measurements can be conducted easily within 360° on a horizontal plane. Together with high-precision GPS, this allows precise location information to be provided during operation. Figure 4 shows a schematic and a photograph of the FISS field measurements based on the multi-use platform.

The FISS system introduced above, together with the multi-use platform, forms the complete FISS system, as shown in Figure 4(b) (GPS not included).

### 2.3. Main Technical Parameters

Various technical parameters are used to characterize FISS, including the spectral range, spectral resolution, spatial resolution, and scanning rate. Table 1 lists the main technical parameters and performance of FISS, most of which are described in Section 3.

#### 2.3.1. Spectral Range and Spectral Resolution

The spectral range of the FISS depends on the dispersing unit and the spectral response range of the CCD camera. The spectral resolution is determined by the entrance slit (nominal width 60 μm) and the size of the CCD photosensitive component. To improve the system SNR and the rate of data acquisition, area array detectors were merged into 3 × 3 units, which reduced the CCD resolution from 1,392 × 1,040 to 464 × 344 (spatial × spectral dimensions). Hence, FISS theoretically has 344 spectral channels. After pixel combination, the photosensitive component size reaches about 20 μm. Due to the 1:1 imaging mechanism of the CCD, the spectral resolution depends mainly on the entrance slit. In the laboratory, the FISS band range and spectral resolution can be determined accurately by spectral calibration using a monochromator (Section 3.1).

#### 2.3.2. FOV and IFOV

The FOV (Field of View) of the FISS, determining the field of view of each track line, is defined by both the effective length of the slit and the focal length of the objective lens as:

(1) tanFOV2=x2f

To the FISS, the effective length of the slit x is 8.8 mm, and the focal length of the objective lens is about 24 mm, hence the FOV is about 21°.

The IFOV (Instantaneous Field of View), reflecting the spatial sampling of FISS, depends on the size of the imaging cell and the focal length of the objective lens as follows:

(2) tanIFOV2=d2f

To the FISS, after 3 × 3 binning, the size of the imaging cell reaches about 19.35 um, so the IFOV is about 0.806 mrad. The IFOV can be also roughly calculated by the ratio of FOV and the number of pixels along the spatial dimension.

#### 2.3.3. Frame Rate

Generally, the frame rate depends on the data transfer rate and the data acquisition mode of the CCD camera. For the CCD camera in the FISS, data were transported through USB 2.0 interfaces with 12-bit data sampling. To avoid image blurring due to asynchrony between data acquisition by the flow mode (camera mode) and the stepper motor, a photo mode was chosen for data acquisition. In practice, this means that each step of the stepper motor issues a synchronous signal that triggers the camera to take a picture. In photo mode, the frame rate of the CCD camera may be up to 20 frames per second. However, this is often set to 10 frames per second in field experiments.

## 3. System Calibration

### 3.1. Spectral Calibration

Using the DK-242 monochromator (two cascaded monochromators, with the exit slit of the first monochromator functioning as the entrance slit of the second) provided by the Anhui Institute of Optics and Fine Mechanics (Chinese Academy of Sciences) and self-developed spectral calibration software, the FISS spectral calibration experiment was carried out in a dark optical laboratory to determine the center wavelength and the FWHM for each spectral channel.

#### 3.1.1. Spectral Range Determination

Before the calibration test, instrument parameters should be set to ensure that the spectral calibration is valid. For the double grating monochromator, the spectral bandwidth of all parallel beam outputs was less than 2 nm, with a step length of 1 nm. For the FISS to be spectrally calibrated, three parameters had to be set: the integration time (100 ms), the size of the aperture (F4), and the CCD cooling temperature (5 °C). Adjusting the relative position between the monochromator and the FISS ensured that the aperture of the FISS was perpendicular to the monochromatic beam, so that it received all beams within a scanning range of ±1°. Illuminated by the DK-242 monochromator, the CCD received two images at each band switch by 1 nm within the spectral region of 400 – 910 nm. The results showed that the FISS will respond to light from the monochromator within 400 – 910 nm (not the same as the centroid wavelengths shown in Table 1).

#### 3.1.2. Number of Spectral Bands

The test method was the same as that described in 3.1.1. On inspection of all the images obtained by FISS in ENVI4.7 software, we found that FISS had 344 spectral channels.

#### 3.1.3. Spectral Resolution Determination

**Figure 5.**
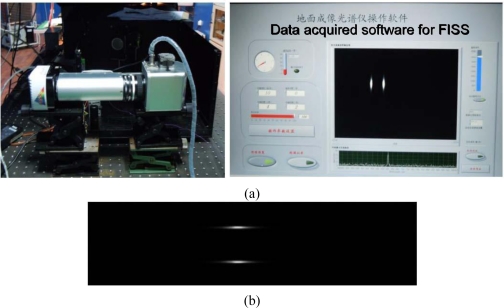
**(a)** Laboratory spectral calibration set-up and **(b)** Imaging results for FISS from 589 nm monochromatic light: two symmetrical narrow lines for each monochromatic light were detected due to the “round trip” of the scan mirror within ±2°.

The test method was the same as that described in 3.1.1. The FISS recorded the imaging data simultaneously, when the monochromator emitted different monochromatic beams in 1-nm stepping intervals within the spectral region 400–910 nm. Figure 5 shows laboratory spectral calibration set-up and the imaging results of FISS for 589 nm monochromatic light. In order to assure the reliability of the laboratory spectral calibration, the scanning angle of the scan mirror of the FISS was set within ±2°, so that two symmetrical narrow lines for each monochromatic light could be obtained [see Figure 5(a)], that is, each monochromatic light was scanned twice by the FISS due to the “round trip” of the scan mirror within ±2°. Eventually, to reduce some system and measurement errors, two independent images for each monochromatic light were employed to spectral calibration by taking the average result of the two “independent” calibration experiments.

Due to the instability of the monochromator and the discrete spectra emitted from it, the spectral response curve of each channel is discrete and has a certain amount of noise. Hence, Gaussian fitting was used for the spectral curve of each channel. The center wavelength and spectral resolution of each channel were then calculated from the function. The Gaussian fitting function used in this paper was:

(3) f(x)=A0e−((x−A1)A2)22+A3

where *A*_0_ is the height of the Gaussian function, *A*_1_ is its center, *A*_2_ is its width (standard deviation), and *A*_3_ is a constant. The center wavelength of each channel can be obtained from *A*_1_ and the spectral resolution (FWHM) can be calculated as:

(4) $$\mathrm{FWHM}=2A_{2}\sqrt{2\;\text{ln}\;(2)}$$

**Figure 6.**
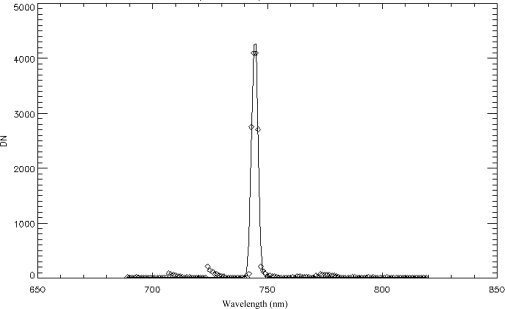
The spectral response (diamonds) for the 231st channel and its Gaussian fitting result (solid line): the diamonds are the actual response (DN) of the 231^st^ channel of the FISS system to the radiance output by the monochromator from 690 to 820 nm; the solid line is the best-fit Gaussian function from which the center wavelength and FWHM channel width can be derived.

**Figure 7.**
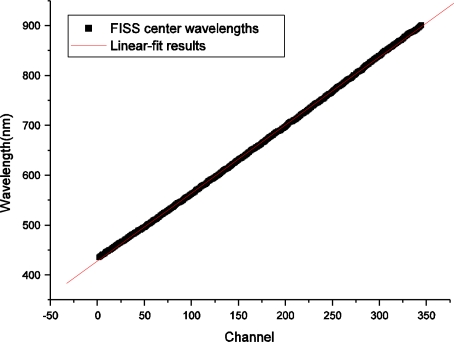
The center wavelength of each channel and linear fitting results: black squares are the actual center wavelengths obtained by calculating *A*_1_ from (3) for all 344 channels, and the red line is the linear fitting result.

Using (3) and (4), the spectral calibration results of each channel were obtained using the IDL 7.1 software. Figure 6 illustrates the spectral response for the 231^st^ channel and its Gaussian fitting result. Figure 7 shows the center wavelength of each channel and the linear fitting results. The center wavelengths determined by indoor spectral calibration were almost perfectly linear, with a correlation coefficient of R = 0.99985.

**Figure 8.**
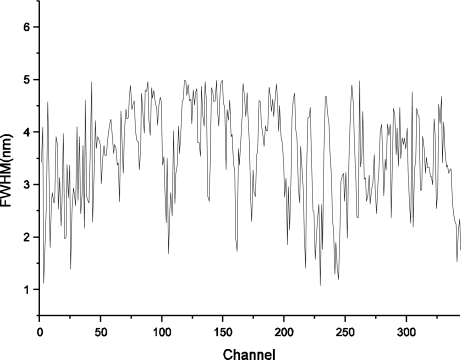
Spectral resolution for all 344 channels of the FISS system.

**Figure 9.**
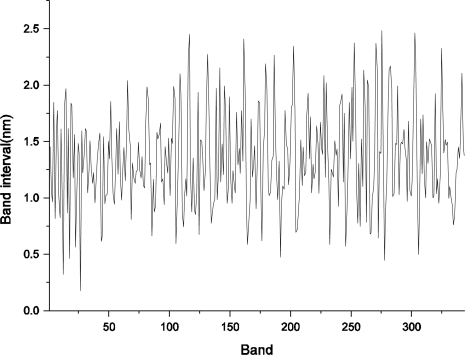
Spectral sampling intervals between adjacent channels of the FISS.

The spectral resolution of FISS for all 344 channels was derived by calculating the FWHM of the Gaussian function for each channel (Figure 8). Statistical analysis was performed to obtain an overall understanding of the calibrated spectral resolution for FISS channels (Table 2). As shown in Figure 8 and Table 2, the FISS system achieved high spectral resolution (better than 5 nm for all spectral channels). Figure 9 shows the spectral sampling intervals calculated from the difference between adjacent spectral channels. The average sampling interval was about 1.4 nm, which may help in further understanding of the FISS system.

### 3.2. Radiometric Calibration

**Figure 10.**
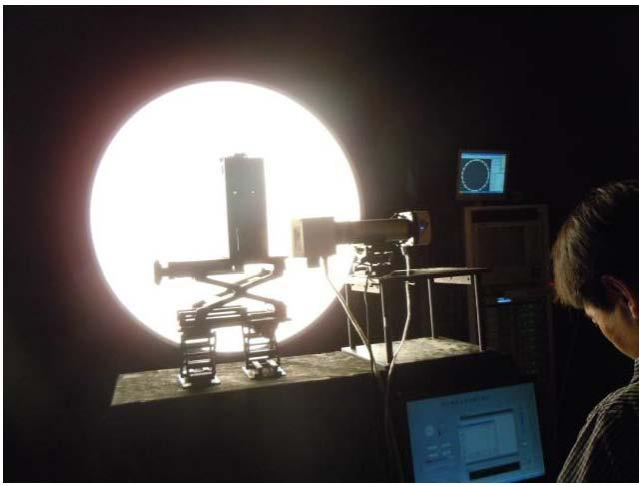
Overview of the calibration experiment: the SVC HR1024 was in front of the integrating sphere (left) and the calibrated FISS system (right). Both were close, and operating simultaneously. The SVC HR1024 was used to cross-calibrate the FISS system, and both were fully illuminated by the integrating sphere.

In this section, we discuss the radiometric calibration and SNR of the FISS system. An overview of the calibration experiment is shown in Figure 10. An integrating sphere was used as an indoor light source to fill the entire field of view of the FISS. A well-calibrated SVC HR1024 spectrometer was placed close by (spectral resolution <3 nm; spectral sampling bandwidth 1.5 nm within the region 350–1,000 nm). By changing the radiance of the integrating sphere (*i.e*., by controlling the number of bright and dark standard lights within the integrating sphere; a total of 17 levels), and changing the aperture size of the FISS optical lens (the aperture size was varied between four levels, F8, F11, F16 and F22), we established a quantitative relationship between the entrance radiance at the pupil of the FISS and the digital number. The SVC HR1024 spectrometer was used to cross-calibrate the FISS system. To simplify the experiment, the integration time and CCD cooling temperature were set to constant values of 30 ms and 10°C, respectively, consistent with the field parameters.

#### 3.2.1. Signal to Noise Ratio (SNR)

The SNR is a key property of the FISS system, but it is complicated to calculate as it depends on many factors [37–39]. Here, a simple SNR model [40] based on images was considered. We calculated the maximum SNR of the FISS system by adjusting the system parameters until the response approached saturation. Thus, the image acquired under “sub-saturated” situations (*i.e.*, aperture size F8; number of bulbs on the integration sphere: 8) was used to compute the system SNR. The dark offset of the FISS system was also measured under the same conditions, with only the light entrance slit blocked. The SNR was calculated as:

(5) $${\mathrm{SNR}}_{i}=\frac{\mu_{i}-{\mathrm{d\_offset}}_{i}}{{\mathrm{d\_offset}}_{i}}$$

where *SNR_i_* is the SNR, *μ_i_* is the mean signal value (DN), and *d_offset_i_* is the mean dark offset (DN) for the i^th^ channel.

**Figure 11.**
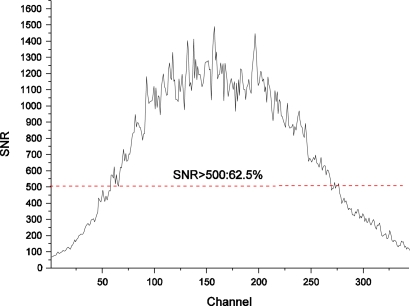
Maximum SNR of the FISS system for each channel: the abscissa is the channel number (band number), while the ordinate is the corresponding SNR calculated. The figure shoes that 215 of the 344 channels (62.5%) responded well, with SNR > 500 (above the dashed red line).

Figure 11 shows the maximum SNR of the FISS system calculated by (5) for each channel. Clearly, the FISS achieves a relatively high SNR, with 62.5% of the total channels greater than 500. The SNRs of the two ends channels, however, are lower.

#### 3.2.2. Radiometric Calibration Process

Assuming a linear system, we used the following equation to accomplish absolute radiometric calibration for the FISS:

(6) Le=a⋅DN+b

where *Le* is the entrance radiance at the pupil, *DN* is the digital number, and *a* (gain) and *b* (offset) are calibration coefficients for each channel. By least squares analysis, *a* and *b* can be calculated using the IDL7.1 software.

**Figure 12.**
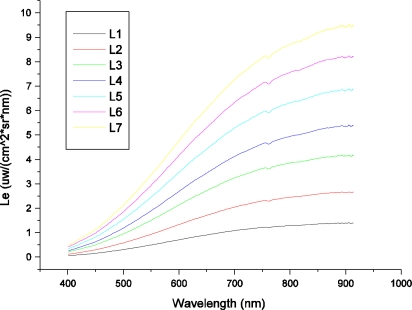
Spectral radiance curves for seven levels obtained by the SVC HR1024 within the spectral region of 400–915 nm. The level number denotes the number of bulbs in the integrating sphere that were switched on.

To facilitate radiometric calibration, an aperture size of F8 was used. To avoid saturating the A/D converters of both the HR1024 spectrometer and the FISS, the radiance emitted by the integrating sphere was set at 7 levels. Figure 12 shows the incidence radiance curves for seven radiance levels obtained by SVC HR1024, which were used to cross-calibrate the FISS.

**Figure 13.**
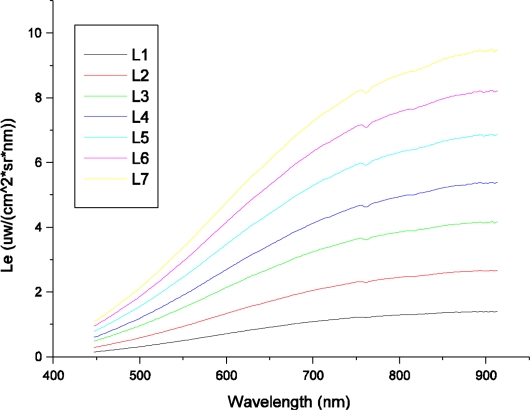
Spectral radiance curves of the FISS interpolated by a multilinear method. The level number denotes the number of bulbs in the integrating sphere that were switched on.

Due to the similar spectral resolution and spectral sampling bandwidth of the SVC HR1024 and FISS within the region of 437–902 nm, we were able to multilinearly interpolate the spectral radiance obtained by HR1024 according to the centroid wavelengths of the FISS. The interpolated results for the seven levels are shown in Figure 13. They were regarded as the radiance of the FISS (*i.e*., Le). In the multilinear interpolation method, one of the centroid wavelengths (F1) of the FISS was compared to the wavelengths of HR1024. If F1 was between the adjacent wavelengths of HR1024, a linear equation was constructed, and the radiance at F1 was calculated through linear interpolation. Hence, 344 linear equations were needed to finish the interpolation.

**Figure 14.**
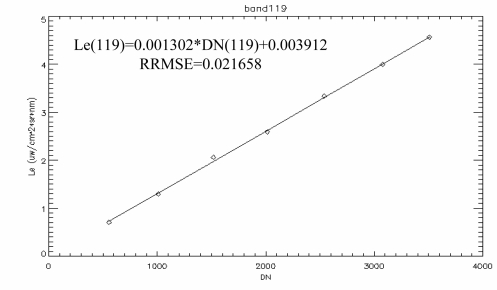
The FISS system radiometric calibration results of band 119.

Using (6), the radiometric calibration coefficients for each channel were calculated. Table 3 shows partial results of the radiometric calibration under the following conditions: the optical lens aperture was set to 8, the CCD cooling temperature was 10 °C, and the integration time was 30 ms. The linear regression results of the 119th band are shown in Figure 14. The data points of band 119 fit the line quite well, with calibration coefficients of a = 0.001302 and b = 0.003912.

#### 3.2.3. Evaluation of Radiometric Calibration Accuracy

The overall laboratory radiometric calibration accuracy depends on various factors, including the precision of calibration standards, the stability of the instruments, and the accuracies of the algorithms used for data processing.

**Figure 15.**
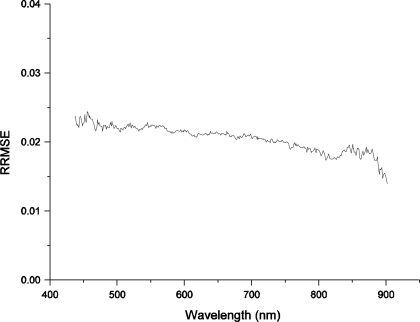
Linear fitting error measured by R-RMSE for each channel (band) of the FISS.

Table 4 shows various independent errors generated during the whole process of laboratory radiometric calibration and the overall calibration accuracy of the FISS calculated from these errors. The error caused by the linear fitting algorithm was measured as the relative root-mean-square error (Relative-RMSE, RRMSE):

(7) RRMSE=∑i=1N((yi−yi)yi)2N−M

where *y_i_* is the measured radiance, *y_i_* is the corresponding fitted value, *N* is the number of radiance levels, and (*N* − *M*) is the number of degrees of freedom. The linear fitting error for each channel is shown in Figure 15.

The overall laboratory absolute radiometric calibration accuracy (*i.e*., the total maximum uncertainty) is better than 5% over the wavelengths covered by the FISS. Therefore, the calibration coefficients can be reliably used in practical applications only if the measurement parameters of the FISS remain the same (aperture size, F8; CCD cooling temperature, 10 °C; integration time, 30 ms).

## 4. Conclusions

Due to their unique advantages in field imaging spectral data acquisition, field imaging spectrometers have attracted interest from researchers and scientists worldwide. Many companies and institutions specialize in the development of such instruments, such as Spectral Imaging Ltd. (Finland), Resonon Inc. (USA), and Surface Optics Corporation (USA). However, before the FISS, no field imaging spectrometer built with a cooling area CCD had been developed in China. This paper introduced the basic principle of the FISS, its structural design, and main technological parameters. To evaluate the performance of the FISS and facilitate practical applications, the sensor was radiometrically and spectrally calibrated in a precise optics laboratory. The FISS system covers a wide spectral range (437–902 nm), sampled by 344 channels, which may detect subtle variations of surface properties with a spectral resolution of better than 5 nm for each channel. The absolute radiometric calibration accuracy for each band is less than 5%. The high calibration accuracy guarantees that applications with FISS are reliable and valid. In addition, the SNR, a critical parameter for understanding the FISS system, was also measured. The results showed that 62.5% of the FISS channels achieved SNR approaching 500:1 or better. Although high performance was achieved by the FISS sensor, the system should be further optimized to suit the requirements of precise applications. The system calibration accuracy seriously affects the accuracy of various applications. Therefore, further studies are required to develop better radiometric and spectral calibration methods. Moreover, to promote quantitative applications with FISS, a look-up table of radiometric calibration coefficients for varying measurement conditions should be generated and frequently updated.

## Figures and Tables

**Table 1.** Main parameters and performance of FISS.

Number of bands	344	Imaging rate	Maximum 20 frames/s
Spectral range	437–902 nm	Scan field	−20° to +20°
Spectral resolution	Better than 5 nm	Quantitative value	12 bit
Spatial resolution	Maximum < 2 mm	Signal to noise ratio	>500 (60% bands)
Radiance calibration precision in laboratory	Better than 5%	Spectral sampling interval	About 1.4 nm

**Table 2.** Statistical results on the spectral resolution for the 344 FISS channels.

**Mean (nm)**	**Standard deviation (nm)**	**Minimum (nm)**	**Maximum (nm)**
3.56714	0.91462	1.08533	4.99930

**Table 3.** Partial results of absolute radiometric calibration. (Aperture size, F/8; CCD cooling temperature, 10 °C; integration time, 30 ms).

**Band**	**Offset (b)**	**Gain (a)**	**Band**	**Offset (b)**	**Gain (a)**
101	0.002758	0.001250	121	0.003983	0.001300
112	0.002439	0.001281	132	0.001756	0.001376
113	0.002473	0.001275	133	0.002127	0.001385
117	0.004356	0.001300	137	0.002321	0.001395
118	0.004016	0.001304	138	0.001451	0.001398
119	0.003912	0.001302	139	0.000937	0.001408
120	0.003621	0.001303	140	0.001385	0.001412

**Table 4.** FISS absolute radiometric accuracy.

**Error sources**	**The maximum uncertainty (%)**	**Notes**

Calibration accuracy of the HR1024 SVC	3.4	Mainly originating from the uncertainty of standard lamps
Measurement repeatability accuracy of the HR1024 SVC	1.1	Standard error of the mean
Integrating sphere surface uniformity	0.19	Keeping all bulbs (64) on
Integrating sphere angle uniformity	2.1	Relative standard error within ±45°(leave one bulb on)
Integrating sphere instability	0.66	Measurement with 8 hours
Linear fitting errors	2.45	See details in Figure 14
Overall calibration accuracy of FISS	4.86	Root-sum-square, RSS
